# A Systematic Review for Cognitive State-Based QoE/UX Evaluation

**DOI:** 10.3390/s21103439

**Published:** 2021-05-14

**Authors:** Edgar Bañuelos-Lozoya, Gabriel González-Serna, Nimrod González-Franco, Olivia Fragoso-Diaz, Noé Castro-Sánchez

**Affiliations:** Department of Computer Science, TecNM/CENIDET, Morelos 62490, Mexico; gabriel.gs@cenidet.tecnm.mx (G.G.-S.); nimrod.gf@cenidet.tecnm.mx (N.G.-F.); olivia.fd@cenidet.tecnm.mx (O.F.-D.); noe.cs@cenidet.tecnm.mx (N.C.-S.)

**Keywords:** QoE, UX, cognitive states, physiological data, behavioral data, biometric sensors

## Abstract

Traditional evaluation of user experience is subjective by nature, for what is sought is to use data from physiological and behavioral sensors to interpret the relationship that the user’s cognitive states have with the elements of a graphical interface and interaction mechanisms. This study presents the systematic review that was developed to determine the cognitive states that are being investigated in the context of Quality of Experience (QoE)/User Experience (UX) evaluation, as well as the signals and characteristics obtained, machine learning models used, evaluation architectures proposed, and the results achieved. Twenty-nine papers published in 2014–2019 were selected from eight online sources of information, of which 24% were related to the classification of cognitive states, 17% described evaluation architectures, and 41% presented correlations between different signals, cognitive states, and QoE/UX metrics, among others. The amount of identified studies was low in comparison with cognitive state research in other contexts, such as driving or other critical activities; however, this provides a starting point to analyze and interpret states such as mental workload, confusion, and mental stress from various human signals and propose more robust QoE/UX evaluation architectures.

## 1. Introduction

User experience and quality of experience refer to a user and his/her experience with an application, product, or service, UX from the perspective of understanding and interpreting user’s perceptions and answers [1] and QoE based on the degree of the user’s delight or annoyance, which turns out to be a quality evaluation [2]. Wechsung and De Moor [3] carried out an analysis of the differences and similarities between both concepts. UX comes from human–computer interaction and is considered more human-centered because of the way observations are captured and interpreted, for example with standardized questionnaires such as the System Usability Scale (SUS) [4] or the Self-Evaluation Manikin (SAM) [5], and non-functional aspects’ analysis such as emotions and other affective states; however, QoE comes from the telecommunications area and is considered more technical because it depends more on technology partly due to its relation to Quality of Service (QoS). Actually, both concepts retain theoretical differences, but in practice, they are converging on some similar evaluation mechanisms. This even suggests consolidating QoE and UX into a broader concept called Quality of User Experience (QUX) [6], which also includes eudaimonic aspects such as the meaningfulness and purpose of use. It was for this reason that this review included papers as QoE/UX regardless of whether their context was one or the other. QUX was not used because it is a construct still under research and definition.

Traditional QoE/UX evaluation mechanisms are subjective by nature because they are based on techniques that depend on users’ reports and evaluators’ analysis influenced by their perception, criteria, and experience, among other personal factors [7,8,9,10]. Several evaluation approaches have been proposed for complementing subjective techniques with quality ratings or mental states inferred from user’s physiological and behavioral data (e.g., [11,12,13,14]). Even though research has been done to interpret the mental states of users when performing certain activities—even critical ones, such as driving, piloting, and air traffic control (e.g., [15,16,17])—the relations between these states and elements of an interface or interaction mechanisms have yet to be identified and adequately represented.

This paper presents a Systematic Literature Review (SLR) carried out to identify and analyze research related to QoE/UX evaluation where cognitive states are interpreted from features of Electroencephalogram (EEG), Galvanic Skin Response (GSR), Electrocardiogram (ECG), and Eye Tracking (ET) (without pupillometry); this includes the machine learning models used, the best results, and the proposed evaluation architectures. Works that analyzed human signal data for searching for correlations between cognitive states and QoE/UX metrics were also considered.

The rest of the paper is structured as follows: The next section presents a background of cognitive states and physiological and behavioral data. Section 3 presents the characteristics of the systematic review protocol. Section 4 describes the final set of articles according to the related topics. Section 5 gives the discussion and findings, and the last section provides the conclusions obtained.

## 2. Background

### 2.1. Mental and Cognitive States

A mental state includes every aspect of the internal state of an organism that could contribute to its behavior or other responses [18]; this includes variables that are present at a given moment such as: thoughts, perceptions, emotions—characterized by valence and arousal—or others that describe cognitive processes.

In particular, the relationship between cognition and emotion has been discussed by other authors [19], finding that their interaction is so complex that it needs to be studied in nuanced terms and with a detailed analysis of the context. Specifically, cognition refers to processes such as memory, attention, language, problem solving, and planning [20], and based on these processes, several states are identified: mental workload, mental stress, and mental fatigue, among others.

The presence of cognitive states can manifest in various ways. For example, it has been found that mental workload can be expressed as a subjective experience, with variations in the task performance and with physiological manifestations [21], or that there are relationships between numerous physical responses with the presence of mental stress, such as agitation, anxiety, sweating, etc. [22].

### 2.2. Physiological and Behavioral Data

Emotions and cognitive states have similarities in terms of the data used for their estimation. These data can be grouped into three categories according to the technologies used to acquire them [23]:

1. based on perception or behavior, including all data from elements of human expression, such as: facial expressions, intonation and voice modulation, body movements, contextual information, etc.;
2. physiological, coming from the subconscious responses of the human body, such as heartbeat, blood pressure, brain activity, etc., related to the central nervous system, the neuroendocrine system, and the autonomous nervous system;
3. subjective, self-reports by individuals about how they perceive their state, being less dependent on technology than the previous two.

This review considered research related to physiological data of the following signals:

- Electroencephalogram, a signal related to electrical activity in the brain, is registered by electrodes attached to the scalp commonly distributed under the 10–20 standard [24]. The power of the signal is due to five rhythms according to the frequency ranges: delta (δ), below 4 Hz; theta (θ), around 5 Hz; alpha (α), around 10 Hz; beta (β), around 20 Hz; and gamma (γ), usually above 30 Hz.
- Electrocardiogram, a signal related to electrical activity generated by the heart muscle, is recorded by placing a set of electrodes on the chest and occasionally on the extremities, depending on the application [24]. A beat has five different waves (P, Q, R, S, and T) that allow determining the heart rate and rhythm.
- Galvanic skin response, also known as Electrodermal Activity (EDA), provides a measurement of the electrical resistance of the skin when placing two electrodes on the distal phalanges of the middle and index fingers, which can increase or decrease according to the variation of sweating of the human body [25].

In the case of behavioral data, research that included eye tracking data was contemplated. Eye tracking is a methodology that, among other features, makes it possible to detect where the user is looking and for how long and the path his/her eyes follow. Eye features can be obtained using electrooculography, video-based analysis, or from specific eye-tracker technology (e.g., [26,27,28]). QoE/UX researchers have widely employed eye-tracking devices that work through cameras and methods to illuminate the eye, identify reflection in the cornea and pupil, and establish the related gaze point [29]. This process allows obtaining features such as fixations and saccades. A fixation is a brief pause of eye movement in a specific area of the visual field. Saccades are quick eye movements between one fixation and another.

Pupil dilation data are considered physiological and directly related to the autonomous nervous system [30]. Due to the restrictions of the research, articles that only used pupillometry were excluded.

## 3. Materials and Methods

A systematic literature review is a methodology to identify, evaluate, and interpret relevant research on a particular topic and responding to specific research questions using a replicable and verifiable process [31].

In this review, recommendations for individual researchers proposed by Kitchenham and Charters [31] were followed, and the SLR protocol and the results were submitted to the supervisors of the research work for criticism and revision. Furthermore, this article was structured according to the guidelines of the Preferred Reporting Items for Systematic Reviews and Meta-Analyses (PRISMA) Statement [32].

### 3.1. Eligibility Criteria

For the purposes of the review, papers had to be written in English and published between 2014 and 2019. Additionally, the following exclusion criteria were defined:

1. papers outside the QoE/UX context;
2. papers recognizing only emotions of the traditional circumplex model of affect [33];
3. papers involving only signal data outside the research scope (fNIRS, fMRI, pupillometry, facial expressions, etc.);
4. papers involving experiments only with disorder-diagnosed participants, for example: autism spectrum disorder.

This review represents an initial effort to develop a QoE/UX evaluation architecture based on the interpretation of users’ cognitive states. The exclusion criteria were mainly constrained by the research scope–context, mental states, signals, and potential users—considering the equipment and current conditions of our laboratory and the time constraints of the review, among others.

The inclusion criteria considered that the papers had to recognize one or more cognitive states with at least one physiological or behavioral signal, including papers on the correlations between those data with QoE/UX metrics or related to evaluation architectures.

### 3.2. Search Strategy

The information sources were: Web of Science, ScienceDirect, SpringerLink, IEEExplore, ACM_DL, arXiv, PubMed, and Semantic Scholar. The execution of the queries was carried out in November 2019.

Four search queries were built with different combinations of keywords taken from four main groups: cognitive states, data from various signals, machine learning, and user experience (Table 1).

The keywords within each group were connected using the OR operator and the groups with the AND operator; the four group combinations for the search queries were:

1. cognitive states AND data AND machine learning AND user experience;
2. cognitive states AND data AND user experience;
3. cognitive states AND user experience;
4. cognitive states AND data AND machine learning.

The last query was not performed in Web of Science due to problems with institutional access to the repository. In Semantic Scholar, issues with exact phrase filters were observed, and consequently, only the first query was carried out. ScienceDirect restricts a maximum of eight connectors in each query, so the most representative keywords of each group were chosen.

### 3.3. Study Selection

The papers resulting from each query were analyzed through the process: (1) duplicate check; (2) evaluation of exclusion criteria based on the title, abstract, and keywords; and (3) evaluation of the eligibility criteria based on the full text. This process was carried out individually and not peer-reviewed; only the results were reviewed by the supervisors of the research work.

The papers that did not meet the eligibility criteria were recorded and labeled as discarded. The papers that passed Stage (3) were tagged as considered and stored using the Mendeley Desktop reference management software.

**Figure 1:**
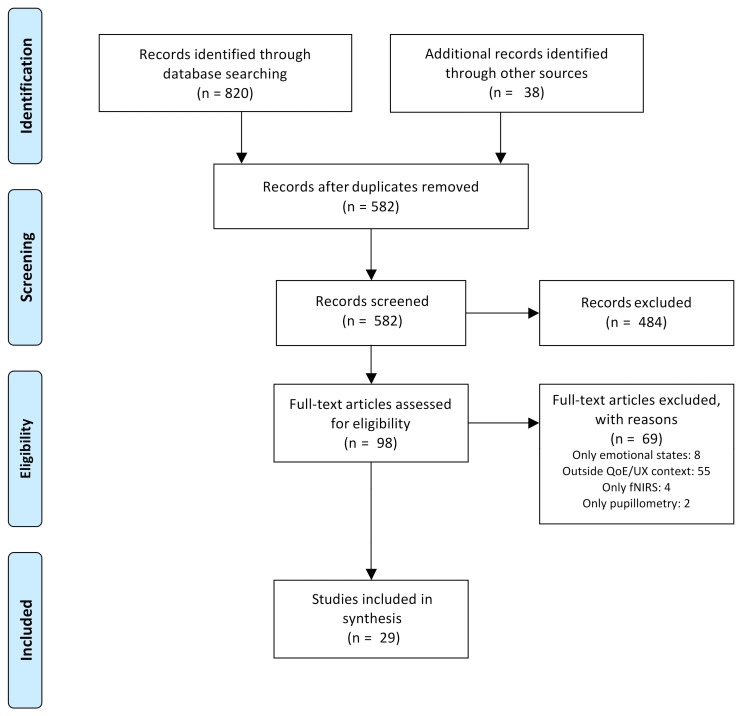
Flow diagram showing the process for papers’ selection.

As shown in Figure 1, a total of 858 records were initially identified. Later, two-hundred seventy-six duplicates were removed, and five-hundred fifty-three records were discarded because they did not meet the eligibility criteria, leaving 29 papers for detailed analysis and data extraction.

### 3.4. Data Extraction

Different data were extracted from the final selection of papers: general data (e.g., authors and institutions of origin, name of the journal or conference), experiment data (e.g., number and characteristics of participants, stimulus, cognitive states, equipment, signals), data related to classification models (e.g., types of machine learning models, features extracted from signals, performance), data related to QoE/UX evaluation architectures (e.g., modules, proposed layers, representation of results), and data related to the obtained results (e.g., findings, conclusions). The registration was initially done on a spreadsheet and later using the Notion software.

## 4. Results

**Figure 2:**
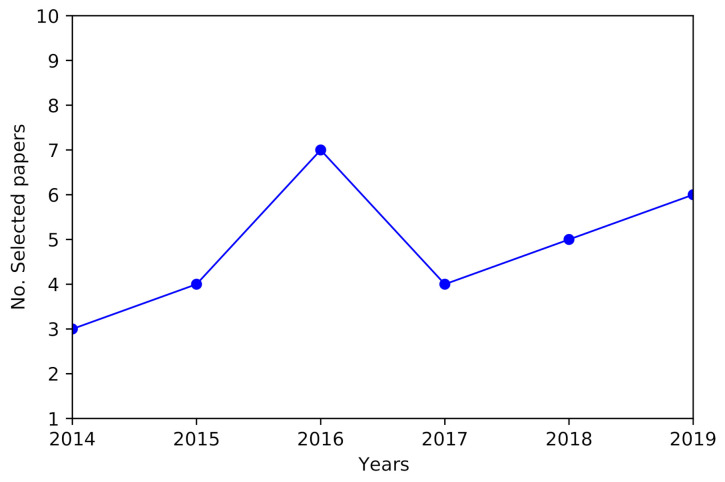
Selected papers by year.

The full-text analysis included 98 papers, of which 56% were discarded because they were outside the QoE/UX context; this gives the insight that related research has been performed, but a lack of research remains in the specific context of this review. Figure 2 presents the chronological distribution of the 29 selected papers; a maximum of seven papers published in 2016 and an average of five papers per year in the period 2015–2019 were observed.

The papers were organized by: (1) research with approaches that refer directly to the QoE/UX evaluation from the classification of cognitive states with machine learning models, (2) papers that contemplate cognitive states, but that present or are part of QoE/UX evaluation architectures, (3) papers that identify correlations between physiological and behavior data with cognitive states and QoE/UX metrics, and (4) other related research.

### 4.1. Classification of Cognitive States

This section considers seven papers related to the classification of cognitive states with machine learning models. They are presented describing independently the classification models and the stimulus used for data capture in the experiments. Table 2 summarizes the characteristics of the described studies in this section, including the model and the best metric reported.

#### 4.1.1. Classification Models

Jimenez-Molina et al. [34] tested models to recognize mental workload with combinations of physiological data and individual ones, having their best results with the Multilayer Perceptron (MLP) that included EEG data; they labeled the physiological data with four classes resulting from a clustering that considered the relationship between the pupil diameter and the mental workload.

In [35], the objective was the classification of engagement, and three studies with various data were carried out: (1) cell phone usage logs in predefined applications and subjective evaluations, (2) daily usage logs and EEG data, and (3) daily usage session logs, context, and demographic data; concluding in the final long-term study that Support Vector Machine (SVM) is the most suitable model in this application compared to others like Random Forest (RF) or AdaBoost.

Frey et al. [36] induced mental workload and assessed attention and performance differences in the task when using a keyboard or touch as the interaction mechanism, extracted EEG data characteristics using spatial filters to reduce from thirty-two channels to six virtual channels, and performed classification tests using Linear Discriminant Analysis (LDA). They found that the performance when using the keyboard was better compared to the use of touch, in addition to the fact that users reported a lower index of mental workload.

Salminen et al. [37] tried the confusion’s prediction using an RF model with eye-tracking data, age, and gender. They used two techniques for data augmentation and found that with the Synthetic Minority Over-sampling Technique (SMOTE) [38], there was better performance, with age as the most influential characteristic. In [39], SMOTE was also used with eye-tracking features, pupil, head position, and clicks; using RF, pupillometry features were determined as the most important in confusion prediction.

On the other hand, in [22], they proposed to infer mental stress based on the pattern of clicks and the gaze. They extracted characteristics from the video from a conventional webcam and mouse, using them with user-dependent and -independent models, using RF at click windows and the logistic classifier at the session level.

Libert and Van Hulle [40] evaluated interest in videos using kNN with EEG characteristics based on entropy and indices of engagement, valence, and activation calculated considering the power of different frequency bands.

#### 4.1.2. Stimulus

One of the investigations [34] presented an experiment with free browsing of a fictitious website, detecting active or transition windows based on gaze fixations at predefined areas of interest.

Mathur et al. [35] conducted their research by recording daily usage data from a cell phone or in predefined applications. This latest study was developed in three months and included participants from various countries due to the feasibility of registering usage logs and obtaining demographics data from the users and context.

Frey et al. [36] performed their pilot study with N-back tasks to induce mental workload and to calibrate the initial models. In their main experiment, participants interacted with a keyboard and touch in a virtual maze game with four difficulty levels.

In [37], modified personal data sheets were used to induce confusion in a journalistic writing assignment. Related to confusion as well, Lalle et al. [39] conducted an experiment with repetitive tasks in an interactive data visualization application where users clicked a button to report confusion at any time.

Huang et al. [22] performed an experiment to induce stress with a software of arithmetic questions that randomly arranged options, adding difficulty levels while displaying a time bar for responses.

In [40], the experiment was carried out with few participants, and it consisted of evaluating interest or omission in observing a set of 45 videos presented one-by-one in three blocks to avoid fatigue.

### 4.2. QoE/UX Evaluation Architectures

Five papers that presented or were part of research that contemplated cognitive states, but with an emphasis on the proposed evaluation architectures were found.

The lean UX-based platform proposed by Hussain et al. [41] aims to support evaluators interpreting observational, physiological, and traditional measures. Its architecture is composed of several layers and includes modules for the recognition of emotions and stress through EEG data analysis and eye tracking, as well as for emotion recognition by analyzing facial expressions, body language, and voice from videos and sounds captured with a webcam and microphone; for these tasks, it used individual classifiers, mostly SVM, with a final merger approach by decision. In addition, it presented modules for the generation of self-report questions and text analysis of responses to detect emotions using an ensemble learning model.

A set of related papers described an approach to assess user experience whose main tool was the physiological heat map [42], which extends the traditional heat gaze map to represent the mental state of the user when interacting with the interface. These maps were validated in an experiment with web pages [43], and although, they were related to visual complexity, it was determined that to maximize its utility, traditional analysis must be integrated (questionnaires, interviews, etc.). On the other hand, they evaluated with expert participants the acceptance and usefulness of UX reports partially completed with images of physiological heat maps, finding that its use is feasible in practice, receiving positive feedback and suggestions for improvement [44]. Furthermore, in [45], they determined the requirements that a UX evaluation tool that considers physiological data and self-reports must meet, highlighting the need to automate data processing and deliver useful results in a timely manner for software development teams that follow agile methodologies, explaining that their proposal is at Technology Readiness Level 6 (TRL 6) and is compatible with commercial devices of data acquisition.

### 4.3. Correlations with Cognitive States and QoE/UX Metrics

This section describes twelve research works where correlations of the different physiological and behavioral signals with cognitive states and diverse QoE/UX metrics were sought. Table 3 summarizes the characteristics of the described studies in this section.

Chai et al. [46] investigated the relationship of frontal alpha EEG asymmetry with experience and difficulty in the task when interacting with a set of mobile applications, not finding meaningful correlations. In [47], the relationship between eye-tracking metrics with self-efficacy, risk, ease-of-use, and usefulness perception in tasks with a software assistant was sought. Various correlations were found, the strongest one being between the perceived ease-of-use and the number of fixations that turn into clicks, providing a guideline for considering the interaction mechanisms with the analysis of inherent signals to humans. In another article [48], the relationship of GSR characteristics with performance metrics was analyzed, identifying that the tasks with a lower rate of completion have a non-significant tendency to cause higher GSR values and a significant correlation between attractiveness, efficiency, dependability, and novelty with GSR data. On the other hand, the usability of a web application was evaluated looking for correlations between subjective questionnaires, EEG, and emotions through facial expressions [49], concluding that EEG measurements are necessary since it was observed that the decrease in motivation was not reflected in the self-reports, but in the increase of brain activity.

**Table 3:** Summary of research of correlations with cognitive states and QoE/UX metrics.

Ref.	Year	Objective	No. of Subjects (Female/ Male)	Stimulus	Data
[46]	2014	Correlations between frontal alpha EEG asymmetry, experience and task difficulty	20 (10F/10M)	Mobile application tasks	Self-report; EEG
[48]	2014	Correlations between GSR and task performance metrics	20 (10F/10M)	Mobile application tasks	Self-report; GSR, blood volume pulse, hear rate, EEG, and respiration
[50]	2014	Correlations between quality perception, brain activity, and ET metrics	19 (11F/8M)	Videos	EEG and ET (with pupillometry)
[51]	2015	QoE evaluation	32 (5F/27M)	Online game	Self-report; EEG
[52]	2015	EEG power analysis during tasks with cognitive differences	30 (20F/10M)	Two-Picture cognitive task and video game	EEG, screen, and frontal videos
[53]	2015	Flow state analysis based on engagement and arousal indices	30 (20F/10M)	Video game	EEG, screen and frontal videos
[54]	2016	Sleepiness analysis	12 (3F/9M), 24 (8F/16M)	Videos	1st study: self-report, EEG, electrooculogram (EOG); 2nd study: self-report, EEG, GSR, ECG, and electromyogram (EMG)
[55]	2017	Cognitive load, product sorting, and users’ goal analysis	21 (10F/11M)	Online shopping tasks	EEG
[47]	2017	Correlations between ET, acceptance and perception	10 (7F/3M)	Database creation assistant	Self-report; ET (with pupillometry), clicks, and screen video
[56]	2018	Visual attention and task performance analysis	38 (not indicated)	Online shopping tasks	ET
[57]	2019	Analysis of the attitude towards a website considering visual attention, cognitive load, product type, and arithmetic complexity	38 (17F/21M)	Online shopping tasks	Self-report; ET (with pupillometry)
[49]	2019	Usability evaluation	30 (15F/15M)	Website tasks	Self-report; screen and frontal videos, mouse and keyboard usage logs, EEG

Arndt et al. [50] analyzed the perception of quality in video fragments using EEG and eye-tracking data, including pupillometry. They observed that pupil dilatation had a great influence due to the use of a visual stimulus, as well as that the alpha EEG activity decreased as the quality’s level decreased, contrary to other studies with a longer stimulus where the participant only had to observe and not evaluate. In another work [51], the QoE evaluation of an online game using standardized questionnaires and EEG measurement was performed, and they found that visual quality was reflected in all the questionnaires applied. Although fatigue’s effect was observable in the physiological data, it was less pronounced as the game time passed. In another investigation [54], tests were performed to analyze sleepiness caused by poor video quality, finding that what was reported by the participants was represented in the EEG data, in particular by alpha waves, inferring that low quality leads to a higher cognitive load and fatigue and a decrease in attention during long-time stimulus.

In [52], significant increases in beta and gamma EEG power were found during relevant events in a platform game compared to normal game events and with another cognitive task. McMahan et al. [53] also evaluated task engagement and arousal using calculated indices from the bands of EEG power and established thresholds and a set of rules to define a flow or immersion model in the platform game.

Desrochers et al. [57] evaluated consumers’ attitudes towards an online site for grocery shopping considering two types of products and tasks of different arithmetic complexity. They obtained visual attention and cognitive load through the analysis of the fixations and pupil’s diameter, respectively, finding that attention toward the product images influenced the attitude towards the site differently depending on the characteristics of the task and on the related cognitive load. In other research work, Juanéda et al. [56] also used fixations to measure attention on a focal product and on similar or dissimilar distractors in close or far away positions. They found that individuals pay less attention to the focal product when distractors are close, becoming more accentuated when distractors are not similar; however, similar distractors had a positive impact on the precision in the attention’s evaluation responses. In other related research, Mirhoseini et al. [55] hypothesized that the user experiences less cognitive load when the method of product sorting is in accordance with the search goal. The cognitive load measurement was interpreted from EEG data with Event-Related Potentials (ERPs), particularly from the P300 component.

### 4.4. Other Related Research

Engelke et al. [58] found that although there is a consensus that multimodal approaches are necessary to fully understand QoE, there is still a shortage of more and better datasets and mechanisms to make them compatible and to integrate them, in addition to the need to standardize the methodologies for capturing and interpreting physiological measurements. In the specific case of eye tracking, Asan and Yang [59] found that despite the devices providing promising information, their use should be integrated with other evaluation techniques, such as most physiological measurements.

In an investigation [60], two paradigms were highlighted for the analysis of EEG data in the context of QoE: ERP and spectral analysis. ERP, in particular P300, was analyzed with stimuli of different characteristics of quality, observing that it was higher and had earlier occurrence when there were distortions in the stimulus, also finding signs of higher levels of fatigue or drowsiness when there was a reduction in the quality of the stimulus. In the case of video games, they observed that the video quality influences quality perception, player experience, subjective measurements, and EEG alpha band frequency.

On the other hand, Salgado et al. [61] presented the demonstration of a prototype where data from various physiological signals in a wheel chair training task in a virtual reality environment were acquired. The last goal of the research was to start from the recognition of various mental states such as stress, drowsiness, and attention, for the future models’ development to be able to determine the QoE.

In [62], Baig and Kavakli performed a review of the use of physiological signals in multimodal systems. Among other findings, they discovered that poorly designed web pages increase the stress level of the user, that simulations can be used to study the relationship between brain responses and stress levels, or that physiological measurements showed a strong correlation with self-reported data and had the ability to extract underlying facts that cannot be found with traditional methods.

## 5. Discussion

In this review, we identified 29 papers within the context of QoE/UX evaluation related to the recognition of cognitive states and published between 2014 and 2019.

**Figure 3:**
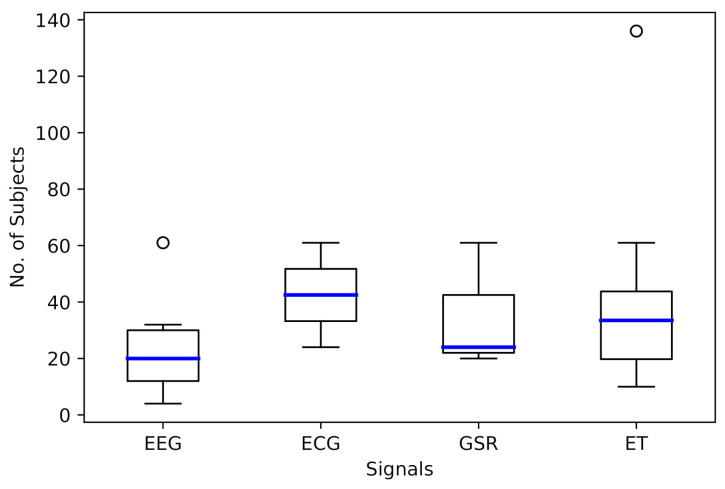
Number of subjects in experiments by signal.

Experiments with different signals and number of participants were identified: EEG data from 4 participants [40], ET data from up to 136 participants [39], or acquiring data from various signals from up to 61 participants [34]. Figure 3 shows the distribution of the number of participants in experiments that collected EEG, ECG, or GSR data and of ET with medians of 20, 42.5, 24, and 33.5, respectively. If more than two signals were used in the experiment, this was considered in an independent way per signal. Atypical values were observed in EEG and ET, denoting that a high number of participants with these signals is not common.

None of the QoE/UX approaches that address the recognition of cognitive states from physiological and behavioral data use deep learning models in some part of the process. Good results have been observed in other contexts with architectures of the autoencoder type (e.g., [63,64]) and of the convolutional type (e.g., [65,66]); however, this can be complicated if the number of participants in the experiments is reduced since the deep learning models require a significant amount of data to take advantage of their potential [67]. Only two of the investigations [37,39] considered techniques such as SMOTE or the Adaptive Synthetic Sampling Approach for Imbalanced Learning (ADASYN) [68] for data augmentation and class balancing. The use of other techniques or models to generate synthetic data was not identified, such as those based on Generative Adversarial Nets (GANs) [69], which are being studied and evaluated in other contexts (e.g., [70,71]).

In general, research does not report the preparation time spent dedicated to each participant. The number of participants may be limited by the type and number of measuring devices that must be configured. On non-invasive EEG devices, in the form of a headband or cap, a greater number of electrodes can imply more time for placement and calibration for each participant. On ET devices, the calibration time is usually shorter, although the lighting conditions in the environment should be considered to a greater extent. In the case of cardiac activity monitoring, a large amount of information and precision are obtained with ECG, whose electrodes are usually placed on the chest or arms, with the disadvantage that these sensors are more intrusive and that their installation requires a stricter protocol compared to those of devices that take heart rate measurements based on PPG. In the case of GSR, sensors are usually placed on the arms, fingers, or forehead, spending little time on its preparation.

To properly select the type and quantity of metering devices used in QoE/UX evaluations, Zeagler’s [72] recommendations can be taken into account for wearable devices and those of Erins et al. [73] in the context of fatigue detection, as the intrusiveness and interference with the task must be minimal, and for this, it is necessary to consider aspects such as the perception of weight, user movement, acceptability, the mobility and availability of the sensor, and susceptibility to the environment, among others. Even before determining the sensors to use, it is necessary to evaluate the convenience of measuring the set of cognitive states proposed in a certain application, and for this, we can initially consider the attributes contributed by Charlton [74] related to sensitivity, intrusion, diagnosis, convenience of measurement, relevance, transferability, and acceptance.

**Figure 4:**
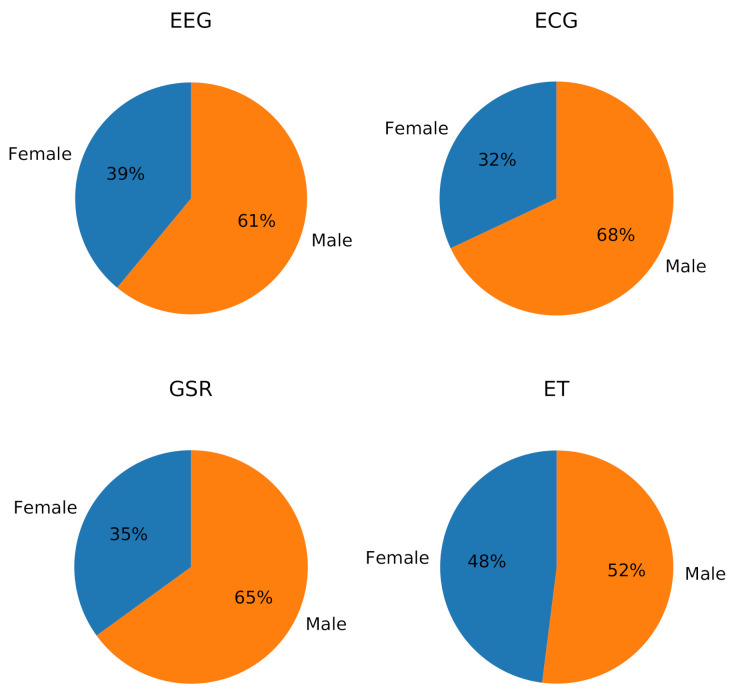
Subject sex ratio in experiments by signal.

In the experiments, the age and sex of the participants were reported, but conclusions related to these aspects were not presented. It has been observed that individual differences given by various factors, such as demographics or experience in the task, can influence physiological and behavioral signals [75]; however, few studies consider these factors (e.g., [76]). Figure 4 shows the proportion of the sex of the participants considering all the experiments related to each signal, and a majority of male participants was observed in EEG, ECG, and GSR, being more equitable in ET; in EEG, the average difference of participants of each sex was 29%, in ECG 36%, in GSR 24%, and in ET 21%. This reaffirms what was found in [62]: standardized experiments are not performed, and the lack of uniformity makes it difficult to establish comparisons between the results.

On the other hand, we identified that the generated datasets are not available for later tests or validations; in this sense, the requirements presented by Mahesh et al. [77] can be generalized to build reference datasets.

The research related to the classification of cognitive states included the following states: mental workload [34,36], engagement [35,40], confusion [37,39], attention [36], and mental stress [22]. Table 2 shows the machine learning models with the best performance. Despite that results with accuracies above 90% (e.g., [34,37]) have been obtained, the classification is based on the interpretation of the user’s cognitive state when responding to the stimulus in general, without studying its relation with specific elements of the interface or the interaction when using an application, adding the difficulties of understanding the relationship of these states with the user’s perception of quality based on the characteristics and changes in the stimulus.

Several papers identified correlations between different physiological and behavioral signals with aspects such as experience and difficulty in the task [46], performance [48,56], and perception of quality [50], among others, and with cognitive processes [50,52] and states such as engagement [53], mental workload [55,57], and attention [56]; however, the usefulness of self-report questionnaires persists and is highlighted, supporting the idea that QoE/UX evaluation mechanisms should be complemented with mixed approaches such as the use of standardized questionnaires and the interpretation of physiological and behavioral signals.

The analyzed evaluation architectures considered several types of sensors and the detection of various mental states: Hussain et al. [41] emphasized the features and independent performance of the models used in each detection module; Courtemanche [42,43,44] highlighted the importance of tools to represent users’ mental states and their usefulness with respect to the evaluators who interpret them and considering the requirements that the industry demands [45]. In general, architectures define modules or layers for data capture and their processing, for the analysis and calculation of metrics, and for the generation and presentation of results, where the process starts with the user performing a task and ends with an expert evaluator interpreting the results and generating or complementing a final report with the findings detected in the test.

The presented review has some limitations. The planning and execution of the search and the selection and analysis of the results were not carried out in a scheme of peer validation, with review and criticism from supervisors, but keeping the intrinsic bias of an individual researcher. The number of analyzed papers was modest given the restrictions to the QoE and UX contexts; the aim was to cover both topics given their similarities in the way of evaluation with physiological and behavioral signals, finding generalized results and not independently detailed.

## 6. Conclusions

This review presented research in the context of QoE/UX evaluation that considered the recognition of cognitive states from EEG, ECG, GSR, and ET data, as well as correlations with QoE/UX metrics, either in individual experiments or as part of evaluation architectures. It showed that cognitive states such as mental workload, stress, and attention, among others, have been analyzed; however, the relation of these states with the elements that build the user experience still need to be studied. The main findings were related to the physiological and behavioral response to the stimulus in general and not to individual components of the interface or interaction. Furthermore, the number and proportion of participants in the experiments and the type and number of measurement devices were varied, and the datasets were not available, limiting the comparability of the results. This review reaffirmed the importance of complementing the evaluations with self-reports and the interpretation of signals from different modalities.

Despite the limitations, this review confirmed the feasibility of these approaches and the need for future studies in order to develop more robust QoE/UX evaluation architectures that allow obtaining results with less subjectivity.

## Figures and Tables

**Table 1:** Groups of search keywords.

Groups	Keywords
Cognitive states	cognitive states, cognitive state
Data	physiological, EEG, GSR, ECG, eye tracking, sensor, multimodal
Machine learning	machine learning, deep learning
User experience	user experience, UX, QoE

**Table 2:** Summary of papers with the classification of cognitive states.

Ref.	Year	Cognitive States	Best Performing Models	No. of Subjects (Female/ Male)	Stimulus	Data
[39]	2016	Confusion	RF, sensitivity 0.61, specificity 0.926	136 (75F/61M)	Data visualization software	Self-report, ET (with pupillometry), clicks
[36]	2016	Mental workload, attention	LDA, accuracy: 92% mental workload and 86% attention	12 (3F/9M)	Virtual maze game	Self-report, EEG, keyboard, and touch behavior
[22]	2016	Mental stress	RF, click-level user-dependent f1-score 0.66; logistic classifier, session-level user-independent f1-score 0.79	20 (7F/13M)	Arithmetic questions software	ET (from video), clicks
[35]	2016	Engagement	SVM, f1-score 0.82	10 (3F/7M), 10 (3F/7M), 130 (34F/96M)	Cell phone usage	1st and 2nd studies: EEG and usage logs; 3rd study: usage logs, context, and demographic data
[34]	2018	Mental workload	MLP, accuracy 93.7%	61 (19F/42M)	Website browsing	EDA, Photoplethysmography (PPG), temperature, ECG, EEG, ET (with pupillometry)
[37]	2019	Confusion	RF, accuracy range 72.6–99.1%	29 (14F/15M)	Personal data sheets	ET, age, gender
[40]	2019	Engagement (as a basis for interest detection)	kNN (k-Nearest Neighbors), average accuracy 80.3%	4 (2F/2M)	Videos	Self-report, EEG

## Abbreviations

The following abbreviations are used in this manuscript:

ADASYN	Adaptive Synthetic Sampling Approach for Imbalanced Learning
ECG	Electrocardiogram
EDA	Electrodermal Activity
EEG	Electroencephalogram
EMG	Electromyogram
EOG	Electrooculogram
ERP	Event-Related Potential
ET	Eye Tracking
GAN	Generative Adversarial Net
GSR	Galvanic Skin Response
kNN	k-Nearest Neighbors
LDA	Linear Discriminant Analysis
MLP	Multilayer Perceptron
PPG	Photoplethysmography
PRISMA	Preferred Reporting Items for Systematic Reviews and Meta-Analyses
QoE	Quality of Experience
QoS	Quality of Service
QUX	Quality of User Experience
RF	Random Forest
SAM	Self-Evaluation Manikin
SMOTE	Synthetic Minority Over-sampling Technique
SVM	Support Vector Machine
SUS	System Usability Scale
TRL	Technology Readiness Level
UX	User Experience
